# Percentile Curves for Body-Mass Index, Waist Circumference, Waist-To-Height Ratio and Waist-To-Height Ratio(Exp) in Croatian Adolescents

**DOI:** 10.3390/ijerph16111920

**Published:** 2019-05-30

**Authors:** Martin Zvonar, Lovro Štefan, Mario Kasović

**Affiliations:** 1 Faculty of Sport Studies dean, University of Brno, 625 00 Brno, Czech Republic; dekan@fsps.muni.cz (M.Z.); Mario.kasovic@kif.hr (M.K.); 2 Faculty of Kinesiology, Department of General and Applied Kinesiology, University of Zagreb, 10 000 Zagreb, Croatia

**Keywords:** secondary-school students, smoothing, centile, anthropometric measures

## Abstract

Purpose: The main purpose of the present study was to establish percentile curves for body-mass index (BMI), waist circumference (WC), waist-to-height ratio (WHtR) and WHtR(exp) in adolescents. Methods: In this cross-sectional study, we recruited 1036 secondary-school students aged 15 to 18 years from eight randomly selected schools (55.3% girls). BMI, WC, WHtR and WHtR(exp) were calculated using standardized measuring protocol. The sex- and age-specific smoothed percentile curves with 5th, 10th, 25th, 50th, 75th, 90th, and 95th percentile for each anthropometric measure were constructed using Cole’s LMS method. Results: In boys, both BMI and WC percentile curves increased by age, yet the 95th percentile curve for WHtR and WHtR(exp) decreased by age. In girls, the 95th percentile curve for BMI remained unchanged through the age of 15 to 18 years, yet the 90th and 95th percentile curves for WC and WHtR decreased by age. Conclusion: This is the first study in Croatia to establish combined BMI, WC, WHtR and WHtR(exp) percentile curves and add some new insight on anthropometric measures in 15- to 18-year-old adolescents.

## 1. Introduction

The prevalence of obesity has risen dramatically in the past several years worldwide, especially in children and adolescents [1]. Specifically, the global age-standardized prevalence of obesity in this age group in 2016 was 5.6% in girls and 7.8% in boys [1]. Obesity is associated with many non-communicable diseases [2] and being overweight or obese in childhood and adolescence often leads to negative health consequences in adulthood [1].

In 2013, the prevalence of obesity in Croatia was 3.5% (2.2% of boys and 4.9% of girls) [3] and those numbers are rising [4]. In addition, data from the Health Behaviour in School Aged Children 2009/2010 survey [5] showed that 23.0% of boys and 10% of girls were classified as overweight. According to the aforementioned survey, special interventions and strategies monitoring and tracking the level of nutritional status in accordance to children’s healthy growth is of extreme importance.

To measure body fat in children and adolescents, instruments such as bioelectrical impedance or magnetic resonance have been constructed, validated, and used [6]. However, such techniques are time-consuming, expensive and in general not practical to use in large epidemiological studies [7]. Thus, body-mass index (BMI), waist circumference (WC) and waist-to-height ratio (WHtR) have been proposed as tools for screening overweight and obesity status [7].

The BMI is calculated from the ratio between weight (in kilograms) and height (in meters) of the participant. It is often used to measure general adiposity and to classify children and adolescents as “underweight”, “normal weight”, “overweight”, or “obese”. Since BMI cut-off values are not the same for children/adolescents and adult population, Cole et al. [8] have established the first age- and sex-specific BMI cut-off values to detect overweight and obesity. Similar values have been created by the World Health Organization [9] and the Centers for Disease Control [10]. However, as highlighted in one study [7], BMI does not differ fat mass from fat-free mass and may lead to misclassification. Previous studies have shown that BMI is not an accurate measurement tool of body fat in children and adolescents, as some other measures like WC and WHtR [11]. 

Both WC and WHtR have been strongly associated with abdominal fatness assessed with imaging tools in children and adolescents [12]. Although there are no specific cut-off points for abdominal obesity measured with WC, a cut-off point of 0.5 for WHtR has previously been proposed as a good indicator of abdominal fatness [13]. Nevertheless, percentile reference charts have been created for both WC and WHtR in European [7,14,15,16], Canadian [17], Asian [18,19,20], and Australian [21] adolescents and have often been used for screening appropriate growth. 

As mentioned before, Croatia has been facing an increase in body weight and BMI in the last two decades [22], especially in adolescents and establishing growth charts can be of crucial value for monitoring and comparing data with expected anthropometric measure parameters [7]. To the best of our knowledge and after an extensive literature review, there have only been two studies conducted among Croatian school-aged children regarding BMI percentile curves [22,23], but no data are known for WC and WHtR. 

Therefore, the main purpose of the present study was to establish percentile curves for body-mass index (BMI), waist circumference (WC), waist-to-height ratio (WHtR) and WHtR(exp) in adolescents aged 15–18 years.

## 2. Materials and Methods 

### 2.1. Study Participants

In this cross-sectional study, participants were secondary-school students. At the first stage, we randomly selected 11 out of 86 secondary-schools (8 grammar and 3 vocational) currently operating in the city of Zagreb. At the second stage, we randomly selected one class representing each grade within the school (from 1st to 4th). Each class had ~25 students. All students were considered healthy and were not affected by diseases. The selection criteria were an active participation in physical education (PE) classes, and an absence of injuries. According to the Croatian Bureau of Statistics for the year 2017 [24], there were 36,350 secondary-school students in total. Our sample size was estimated to be 1030 by using a 95% confidence level and a 3% margin error. At the beginning, we recruited 1247 participants. Of these, 136 did not provide full data and 75 were absent when the study was conducted. Our final sample was based on 1036 secondary-school students (mean ± SD: 16.3 ± 1.1 years, 1.74 ± 0.1 m, 64.7 ± 12.4 kg, 21.3 ± 3.0 kg/m^2^; 55.3% girls). After the selection of each school and class, we contacted physical education teachers to help us organize the study and obtain the approval of the principal. The measurement protocol for the study lasted from January to March 2019. For ≈25 students, it took us 30 minutes in each physical education class to collect the data. Before the study began, all students were familiarized with the aims, hypotheses and benefits of participation in the study. All procedures performed in this study were in accordance with the Declaration of Helsinki and approved by the Institutional Review Board of the Faculty of Kinesiology, University of Zagreb (Ethics code 02/2019). Additionally, all participants and their parents/guardians provided written informed consent for participation in the study.

### 2.2. Anthropometric Measures

Body height was measured to the nearest millimeter in bare or stocking feet with the adolescent standing upright against a stadiometer (Seca, Japan). The result was given in meters. Body weight was measured to the nearest 0.1 kilogram and the participant wore light clothes with no shoes (Seca, Japan). The result was given in kilograms. BMI (kg/m^2^) was calculated as weight (in kilograms) divided by the square of height (in meters). WC was measured for each participant while standing still. We used anthropometric tape placed horizontally midway between the lower rib margin and the iliac crest at the end of normal expiration [25]. WHtR was calculated as WC (in centimeters) divided by the height (in centimeters). The final measure we calculated was WHtR(exp). Previous studies have shown that, during periods of growth, the WHtR retains residual correlation with height, causing the measure to over- or under-adjust for the effect of height at different ages [26]. Therefore, we raised height to a sex- and age-specific power [26]. Specifically, Tybor et al. [26] showed that the optimal exponents were 0.89, 1.11, 1.02, and 0.96 for 15–18-year-old boys and 0.61, 0.62, 0.74, and 0.93 for 15–18-year-old girls.

### 2.3. Data Analysis

Age (without decimal places) and sex were self-reported. The reference 5th, 10th, 25th, 50th, 75th, 90th, and 95th percentiles were constructed for each anthropometric measure, as done in previous studies [7]. One-way analysis of variance (ANOVA) was used to calculate the differences in BMI, WC, WHtR and WHtR(exp) between age and sex. We used Cole’s Lambda, Mu and Sigma (LMS) method, in which the optimal power to obtain normality is summarized by a smooth (L) curve and trends in the mean (M) and coefficient of variation (S) are similarly smoothed [27]. Next, all three curves (L, M and S) are summarized based on the power of age-specific Box–Cox power transformations for normalizing the data [27]. All analyses were performed in Statistical Packages for Social Sciences (SPSS Inc., Chicago, Illinois, USA) and in LMS Chartmaker Pro version (The Institute of Child Health, London, UK).

## 3. Results

Basic descriptive statistics of the study participants are presented in Table 1. In boys, the average value of BMI (F1,3=9.024, *p* < 0.001), WC (F1,3=6.29, *p* < 0.001), WHtR (F1,3=2.97, *p* = 0.032), and WHtR(exp) (F1,3=21.72, *p* < 0.001) significantly increased from the age of 15 to the age of 18. In girls, there were no significant differences in BMI (F1,3=1.38, *p* = 0.249), WC (F1,3=0.46, *p* = 0.707), and WHtR (F1,3=0.46, *p* = 0.711) according to age, except for WHtR(exp) (F1,3=59.17, *p* < 0.001).

**Figure 1:**
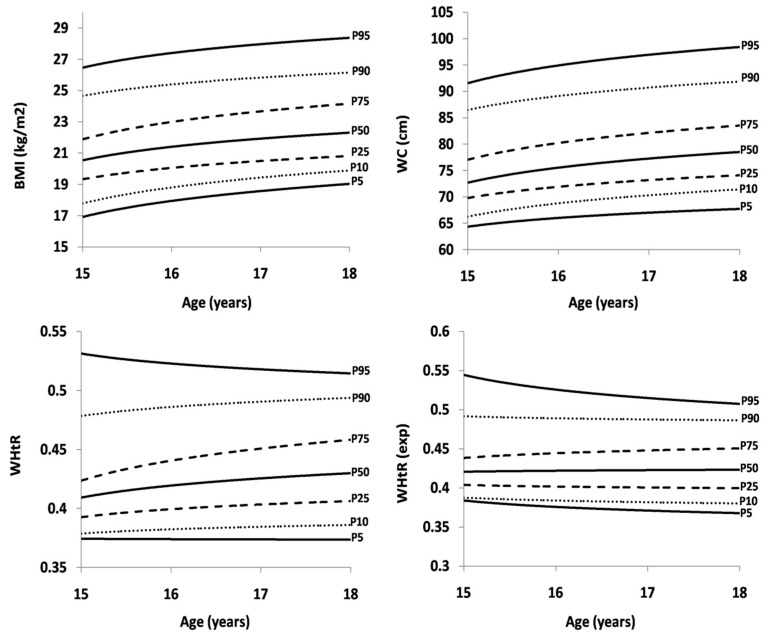
Age-specific reference curves for BMI, WC, WHtR and WHtR^(exp)^ in boys.

**Figure 2:**
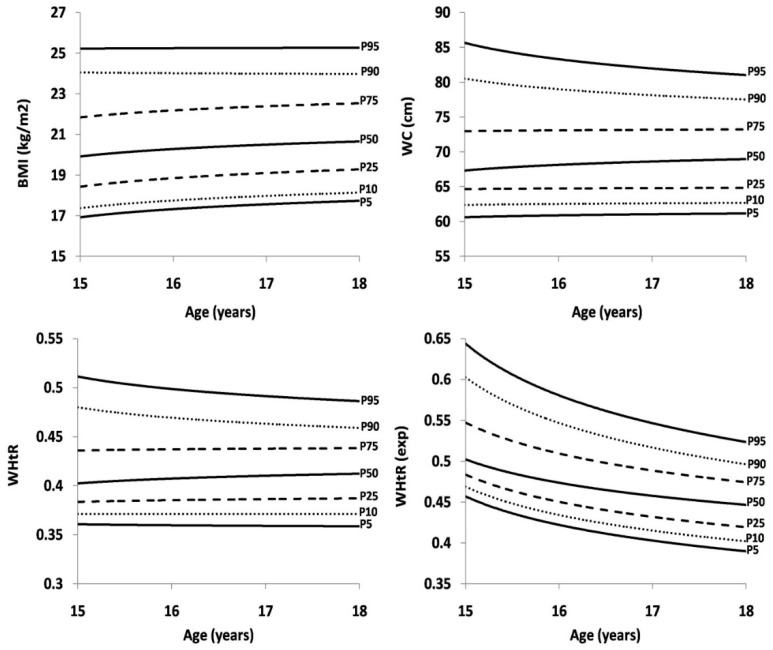
Age-specific reference curves for BMI, WC, WHtR and WHtR^(exp)^ in girls.

Table 2 shows sex- and age-specific percentiles for BMI, WC, WHtR and WHtR(exp). The same values are presented in Figure 1 (boys) and Figure 2 (girls). In boys, there was an increase in all BMI and WC percentile curves between the ages of 15 to 18 years. In WHtR and WHtR(exp), the highest 95th percentile curve slightly decreased from the age of 15 to 18 years, while the 10th, 25th and 50th percentile curves for WHtR(exp) were almost straight, according to age. In girls, the 90th and 95th percentile curves for BMI were straight from the age of 15 to 18 years, while curves from the 5th to the 75th percentile increased. In WHtR, the 90th and 95th percentile curves slightly decreased by age, yet curves from the 5th to the 75th percentile remained straight. A decreasing trend was observed in WHtR(exp) in all percentiles, with the largest drop in the 90th and 95th percentile curve.

## 4. Discussion

The main purpose of the present study was to establish percentile curves for body-mass index, waist circumference, waist-to-height ratio and WHtR(exp) in adolescents. This is the first study examining the aforementioned combined percentile curves in a relatively large sample of Croatian adolescents. These curves represent the first percentile curves to detect and monitor overweight/obesity and abdominal obesity in 15- to 18-year-old adolescents.

Our study showed that boys had higher body-mass index, waist circumference and waist-to-height ratio values, compared with girls. Boys had higher increment in all the aforementioned variables from the age of 15 to 18 years, while values were in general stable in girls. Interestingly, the 90th and 95th percentile curves for body-mass index and waist circumference increased with age, while in girls, those curves were stable for body-mass index and even decreased for waist circumference. Biologically, girls enter puberty much sooner than boys and by the age of 15, anthropometric characteristics become more stable. Boys enter puberty roughly at the age of 13 and their hormonal factors and sexual maturation significantly affect their anthropometric characteristics even after the age of 15 to 16 years.

In the last two decades, the average value of BMI has increased dramatically in Croatian adolescents [22]. Specifically, the largest increase in body weight was in the 16-year-old age group of boys (8.7 kilograms) and in the 11- to 12-year-old age group of girls (5.2 kilograms) [22]. Moreover, Croatian adolescents are facing a major decline in physical activity (PA) [28], which is one of the most common factors influencing BMI. In this study, Štefan et al. [28] showed that the total energy expenditure (TEE) was reduced by 13 kcal/kg/day on average in boys and by 10 kcal/kg/day in girls, while mean daily active energy expenditure (AEE) decreased by 7 kcal/kg/day and 3 kcal/kg/day in boys and girls, respectively. Similarly, the amount of moderate PA declined by 49 min/day in boys and 21 min/day in girls, while vigorous PA was cut by 14 min/day and 3 min/day in boys and girls, respectively [28]. On the other hand, a study by Milosavljević et al. [29] showed that breakfast skipping was a common habit, especially in boys, and fad dieting was more common in overweight adolescents. In addition, the same study confirmed a high consumption of sweets and soft drinks [29], pointing out that both PA and diet play an important role in changing BMI and weight status in general [30]. 

Previous studies have shown that defining overweight and obesity status in adolescents is still a difficult task [31]. By comparing Croatian BMI reference curves with the United States Centers for Disease Control and Prevention (2000) [9,10] and the World Health Organization (2007) [9], Jureša et al. [23] showed that the 5th percentile in boys and girls was similar in both international reference values, while the 50th, 85th and 95th percentiles in boys showed an increase in all ages, while an increase until the age of 14 was observed in girls, after which a decrease in average BMI remained. The same study concluded that national percentile curves should be established for each county, so that international standards can be used for comparable data in different populations [23].

Both WC and WHtR have been previously associated with abdominal fatness assessed with imaging tools in children and adolescents [12]. Moreover, both measures represent simple clinical tools of central obesity [32]. Our established percentile curves for WC and WHtR are in line with previous studies in Greek [32], Bulgarian [33], and Indian [34] adolescents. Specifically, Galcheva et al. [33] showed an upward trend in all WC percentile curves in boys, yet in girls all percentile curves tended to flatten. Similar trends were observed in Indian boys, yet the 50th to 95th percentile curve also tended to flatten after the age of 15 years in girls [34]. Bacopoulou et al. [31] presented similar WHtR percentile curves for Greek adolescents. Although not the main purpose of our study, we calculated the relationship between WHtR and overweight/obesity status (≥85 percentile) using receiver operating curve (ROC) and showed that the optimal cut-off point for boys was 0.44 and 0.42 for girls, pointing out that age- and sex-specific cut-offs should be established nationally. It is worthwhile noticing that WC can be measured in different sites [7], also changing the WHtR value and, as proposed by one previous study [7], international standardized methodology measuring WC should be established in order to compare mean and percentile values between different populations of children and adolescents. Since BMI cannot distinguish between fat mass and fat-free mass [7], and can potentially misclassify risk for overweight/obesity, both WC and WHtR should also be taken into account when screening for general and abdominal obesity. 

In conclusion, this is the first study establishing combined BMI, WC, WHtR and WHtR(exp) percentile values in a sample of Croatian 15- to 18-year-old adolescents. In boys, all percentile curves for body-mass index, waist circumference and waist-to-height ratio increased, except for the waist-to-height ratio 95thpercentile curve, which decreased. In girls, body-mass index, waist circumference and waist-to-height ratio percentile curves remained relatively stable, except for the 90th and 95th percentile curves for waist-to-height ratio, which decreased by age. All percentile curves forwaist-to-height ratio(exp) similarly decreased by age. Although we conducted the study on a large sample of urban secondary-school students and the data cannot be used for rural or mixed population, our results can be used for comparisons in adolescents with different nationalities.

## Figures and Tables

**Table 1:** Basic descriptive statistics of the study participants (Croatia, 2019).

Sex	Age	*n*	BMI (kg/m^2^)		WC (cm)		WHtR		WHtR^(exp)^	
			Mean	SD	Mean	SD	Mean	SD	Mean	SD
**Boys**	15	117	21.1	3.0	75.0	8.3	0.42	0.05	0.45	0.05
	16	105	21.5	2.6	76.8	8.2	0.43	0.04	0.40	0.04
	17	143	22.2	3.2	78.5	10.6	0.43	0.06	0.42	0.05
	18	98	23.2	3.5	80.4	10.3	0.44	0.06	0.45	0.06
**Girls**	15	182	20.4	3.2	70.0	8.0	0.42	0.05	0.51	0.06
	16	172	20.8	2.4	69.6	8.4	0.41	0.05	0.50	0.06
	17	117	20.6	2.2	69.1	6.6	0.41	0.04	0.47	0.04
	18	102	21.0	2.4	70.1	5.8	0.41	0.03	0.43	0.04

**Table 2:** Sex- and age-specific percentiles body-mass index (BMI), waist circumference (WC) and waist-to-height ratio (WHtR) and WHtR^(exp)^ (Croatia, 2019).

Measure	Sex	Age	*n*	Percentile						
				P5	P10	P25	P50	P75	P90	P95
**BMI (kg/m^2^)**	**Boys**	15	117	16.9	17.7	19.6	20.7	22.1	24.9	26.7
		16	105	18.0	19.1	19.6	21.2	22.5	25.0	26.8
		17	143	18.2	19.2	20.2	21.7	23.5	25.6	28.2
		18	98	19.2	19.4	21.3	22.6	24.6	26.5	28.5
	**Girls**	15	182	16.7	17.2	18.4	19.9	21.8	24.1	25.2
		16	172	17.8	18.1	19.0	20.3	22.3	24.3	25.6
		17	117	17.4	17.9	20.4	22.4	23.2	24.5	25.9
		18	102	17.6	18.1	19.3	20.7	22.7	24.5	25.7
**WC (cm)**	**Boys**	15	117	65.0	66.9	70.0	73.0	77.0	86.2	93.2
		16	105	65.0	68.0	72.0	75.0	80.0	90.0	92.7
		17	143	66.0	69.0	72.0	77.0	83.0	90.0	94.0
		18	98	69.0	72.9	75.0	79.0	83.0	92.0	102.0
	**Girls**	15	182	60.2	62.3	65.0	68.0	73.0	80.0	84.0
		16	172	62.0	63.0	64.0	67.0	73.7	80.0	87.0
		17	117	60.5	61.9	65.0	68.0	71.5	78.2	81.2
		18	102	61.0	63.0	65.0	70.0	74.2	77.0	80.0
**WHtR**	**Boys**	15	117	0.37	0.38	0.39	0.40	0.42	0.48	0.54
		16	105	0.38	0.38	0.40	0.42	0.44	0.49	0.51
		17	143	0.37	0.38	0.40	0.42	0.44	0.49	0.51
		18	98	0.37	0.39	0.41	0.43	0.46	0.49	0.53
	**Girls**	15	182	0.36	0.37	0.38	0.41	0.43	0.48	0.50
		16	172	0.36	0.37	0.38	0.40	0.44	0.47	0.52
		17	117	0.36	0.37	0.39	0.41	0.43	0.46	0.49
		18	102	0.36	0.37	0.39	0.42	0.44	0.46	0.48
**WHtR^(exp)^**	**Boys**	15	117	0.40	0.40	0.42	0.44	0.46	0.50	0.58
		16	105	0.35	0.36	0.38	0.40	0.41	0.46	0.47
		17	143	0.37	0.37	0.39	0.42	0.44	0.48	0.50
		18	98	0.38	0.40	0.42	0.44	0.47	0.50	0.54
	**Girls**	15	182	0.45	0.46	0.47	0.49	0.53	0.58	0.61
		16	172	0.44	0.45	0.47	0.49	0.54	0.58	0.63
		17	117	0.41	0.43	0.44	0.47	0.49	0.53	0.55
		18	102	0.38	0.38	0.40	0.43	0.46	0.47	0.50
